# Homology-based annotation of non-coding RNAs in the genomes of *Schistosoma mansoni *and *Schistosoma japonicum*

**DOI:** 10.1186/1471-2164-10-464

**Published:** 2009-10-08

**Authors:** Claudia S Copeland, Manja Marz, Dominic Rose, Jana Hertel, Paul J Brindley, Clara Bermudez Santana, Stephanie Kehr, Camille Stephan-Otto Attolini, Peter F Stadler

**Affiliations:** 1 Bioinformatics Group, Department of Computer Science and Interdisciplinary Center for Bioinformatics, University of Leipzig, Härtelstraße 16-18, D-04107 Leipzig, Germany; 2 Department of Microbiology, Immunology & Tropical Medicine, George Washington University Medical Center, 2300 I Street, NW, Washington, DC 20037, USA; 3 Memorial Sloan-Kettering Cancer Center, Computational Biology Department, 1275 York Avenue, Box # 460, New York, NY 10065, USA; 4 Max Planck Institute for Mathematics in the Sciences, Inselstrasse 22, D-04103 Leipzig, Germany; 5 Fraunhofer Institute for Cell Therapy and Immunology, Perlickstraße 1, D-04103 Leipzig, Germany; 6 Santa Fe Institute, 1399 Hyde Park Rd, Santa Fe, NM 87501, USA; 7 Institute for Theoretical Chemistry, University of Vienna, Währingerstraße 17, A-1090 Wien, Austria; 8 Department of Biology, National University of Colombia, Carrera 45 No. 26-85, Bogotá, D.C., Colombia

## Abstract

**Background:**

Schistosomes are trematode parasites of the phylum Platyhelminthes. They are considered the most important of the human helminth parasites in terms of morbidity and mortality. Draft genome sequences are now available for *Schistosoma mansoni *and *Schistosoma japonicum*. Non-coding RNA (ncRNA) plays a crucial role in gene expression regulation, cellular function and defense, homeostasis, and pathogenesis. The genome-wide annotation of ncRNAs is a non-trivial task unless well-annotated genomes of closely related species are already available.

**Results:**

A homology search for structured ncRNA in the genome of *S. mansoni *resulted in 23 types of ncRNAs with conserved primary and secondary structure. Among these, we identified rRNA, snRNA, SL RNA, SRP, tRNAs and RNase P, and also possibly MRP and 7SK RNAs. In addition, we confirmed five miRNAs that have recently been reported in *S. japonicum *and found two additional homologs of known miRNAs. The tRNA complement of *S. mansoni *is comparable to that of the free-living planarian *Schmidtea mediterranea*, although for some amino acids differences of more than a factor of two are observed: Leu, Ser, and His are overrepresented, while Cys, Meth, and Ile are underrepresented in *S. mansoni*. On the other hand, the number of tRNAs in the genome of *S. japonicum *is reduced by more than a factor of four. Both schistosomes have a complete set of minor spliceosomal snRNAs. Several ncRNAs that are expected to exist in the *S. mansoni *genome were not found, among them the telomerase RNA, vault RNAs, and Y RNAs.

**Conclusion:**

The ncRNA sequences and structures presented here represent the most complete dataset of ncRNA from any lophotrochozoan reported so far. This data set provides an important reference for further analysis of the genomes of schistosomes and indeed eukaryotic genomes at large.

## Background

**Non-coding RNA **(ncRNA) plays a crucial role in gene expression regulation, cellular function and defense, and disease. Indeed, in higher eukaryotes, most of the genomic DNA sequence encodes non-protein-coding transcripts [1]. In contrast to protein-coding mRNAs, ncRNAs do not form a homogeneous class. The best-characterized subclasses form stable basepairing patterns (secondary structures) that are crucial for their function. This group includes the well-known tRNAs, catalytically active RNAs such as rRNA, snRNAs, RNase P RNA, and other ribozymes, and regulatory RNAs such as microRNAs and spliceosomal RNAs that direct protein complexes to specific RNA targets. Much less is known about long mRNA-like ncRNAs, which are typically poorly conserved at the level of both sequence and structure.

Most non-vertebrate genome projects have put little emphasis on a comprehensive annotation of ncRNAs. Indeed, most non-coding RNAs, with the notable exception of tRNAs and rRNAs, are difficult or impossible to detect with BLAST in phylogenetically distant organisms. Hence, ncRNA annotation is not part of generic genome annotation pipelines. Dedicated computational searches for particular ncRNAs, for example, RNase P and MRP [2,3], 7SK RNAs [4,5], or telomerase RNA [6,7], are veritable research projects in their own right. Despite best efforts, ncRNAs across the animal phylogeny remain to a large extent uncharted territory.

The main difficulty with ncRNA annotation is poor sequence conservation and indel patterns that often correspond to large additional "expansion domains". In many cases, the secondary structure is much better conserved than the primary sequence, providing a means of confirming candidate ncRNAs even in cases where sequence conservation is confined to a few characteristic motifs. Secondary structure conservation can also be utilized to detect homologs of some ncRNAs based on characteristic combinations of sequence and structure motifs using special software tools designed for this purpose.

In [8] we described a protocol for a more detailed homology-based ncRNA annotation than what can be achieved with currently available automatic pipelines. Here, we apply this scheme to the genome of *S. mansoni*, and by comparison with the newly sequenced *S. japonicum *genome, identify ncRNAs in both of these clinically important schistosomes.

Schistosomes belong to an early-diverging group within the Digenea, but are clearly themselves highly derived [9-11]. The flatworms are a long-branch group, suggesting rapid mutation rates (see [12]).

**Schistosome genomes **are comparatively large, estimated to be over 350 megabase pairs, and perhaps as high as 400 megabase pairs, for the haploid genome of *S. mansoni *and *S. japonicum *[13-15]. The other major schistosome species parasitizing humans probably have a genome of similar size, based on the similarity in appearance of their karyotypes [16]. These large sizes may be characteristic of platyhelminth genomes in general: the genome of *Schmidtea mediterranea *is even larger, with the current genome sequencing project reporting a size of ~480 million base pairs [17]http://genome.wustl.edu/genomes/view/schmidtea_mediterranea/.

Genome sequencing of the seven autosomes and the pair of sex chromosomes of *S. mansoni *with about 8× coverage has lead to a genome assembly comprising 5,745 scaffolds (> 2 kb) covering 363 Mb [13,14,18]. Similarly, shotgun sequencing of *S. japonicum *with coverage of 5.4× decoded 397 Mb of sequence [15]. These form about 25,000 scaffolds. Albeit both genome projects did not lead to complete finished genomes, we therefore know at least 90-95% of the genomic DNA sequences of *S. japonicum *and *S. mansoni*, respectively.

The protein-coding portion of the *Schistosoma *genomes have received much attention in recent years. Published work includes transcriptome databases for both *S. japonicum *[19] and *S. mansoni *[20], microarray-based expression analysis [21], characterization of promoters [22,23], and physical mapping and annotation of protein-coding genes from both the *S. mansoni *and *S. japonicum *genome projects [18]. Recently, a systematic annotation of protein-coding genes in *S. japonicum *was reported [24]. In contrast to other, better-understood, parasites such as *Plasmodium *[25], however, not much is known about the non-coding RNA complement of schistosomes. Only the spliced leader RNA (SL RNA) of *S. mansoni *[26], the hammer-head ribozymes encoded by the SINE-like retrotransposons Sm-*α *and Sj-*α *[27,28], and secondary structure elements in the LTR retrotransposon *Boudicca *[29] have received closer attention. Ribosomal RNA sequences have been available mostly for phylogenetic purposes [30], and tRNAs have been studied to a limited degree [31].

The wealth of available ESTs, in principle, provides a valuable resource for ncRNA detection. Since mostly poly-A ESTs have been generated, it is not surprising that most ESTs have been attributed to protein-coding genes [32]. The large evolutionary distance, with 55% of the genes without homologs outside the genus [13,18], makes it hard or even impossible to reliably distinguish ESTs of putative mRNA-like ncRNAs from non-coding portions of protein-coding transcripts.

In this contribution we therefore focus on a comprehensive overview of the evolutionary conserved non-coding RNAs in the genomes of *S. mansoni *and *S. japonicum*. We discuss representatives of 23 types of ncRNAs that were detected based on both sequence and secondary structure homology.

## Results and discussion

Structure and homology-based searches of the schistosome genomes revealed ncRNAs from 23 different RNA categories. Table 1 lists these functional ncRNA categories, the number of predicted genes in each category, and references associated with each RNA type. Supplementary fasta files containing the ncRNA genes, bed files with the genome annotation, and stockholm-format alignment files can be accessed at http://www.bioinf.uni-leipzig.de/Publications/SUPPLEMENTS/08-014.

**Table 1:** Summary of homology-based RNA annotations from the sequenced genomes of *S. mansoni *and *S. japonicum*.

RNA class	Functional Category	***S. man***.	***S. jap***.	Related reference(s)
7SK	Transcription regulation	(1)	0	This study

Hammerhead ribozymes	Self-cleaving	> 38, 000	> 5, 000	[27]

miRNA	Translation control	8	7	[109], this study

potassium channel motif	RNA editing	9	3	[65]

RNase MRP	Mitochondrial replication, rRNA processing	(1)	(1)	This study

RNase P	tRNA processing	1	1	This study

rRNA-operon	Polypeptide synthesis	80-105	50-280	[39], this study
5S rRNA	Polypeptide synthesis	21	1-13	This study

SL RNA	*Trans*-splicing	6-48	1-9	[26], this study

SnoRNA U3	Nucleolar rRNA processing	1	1	This study

SRP	Protein transportation	12	4+1	This study

tRNA	Polypeptide synthesis	663	154	This study

U1	Splicing	3-34	2-6	[44], this study
U2	Splicing	3-15	1-63	[44], this study
U4	Splicing	1-19	1-6	[44], this study
U5	Splicing	2-9	1-24	[44], this study
U6	Splicing	9-55	2-12	[44], this study
U11	Splicing	1	1	This study
U12	Splicing	1-2	0-1	[44], this study
U4atac	Splicing	1	1	This study
U6atac	Splicing	1	1	This study

U7	Histone maturation	0	(2)	This study

Where are range of numbers is given, it remains uncertain whether multiple copies in the genomic DNA are true copies of the gene or assembly artifacts.

### Transfer RNAs

Candidate tRNAs were predicted with tRNAscan-SE in the genomes of *S. mansoni*, *S. japonicum *and *S. mediterranea *(a free-living platyhelminth, used for comparison). After removal of transposable element sequences (see below), tRNAscan-SE predicted a total of 713 tRNAs for *S. mansoni *and 739 for *S. mediterranea*, while 154 tRNAs were found in the *S. japonicum *sequences. These included tRNAs encoding the standard 20 amino acids of the traditional genetic code, selenocysteine encoding tRNAs (tRNAsec) [33] and possible suppressor tRNAs [34] in all three genomes. The tRNAsec from schistosomes has been characterized, and is similar in both size and structure to tRNAsec from other eukaryotes [35].

**Figure 1:**
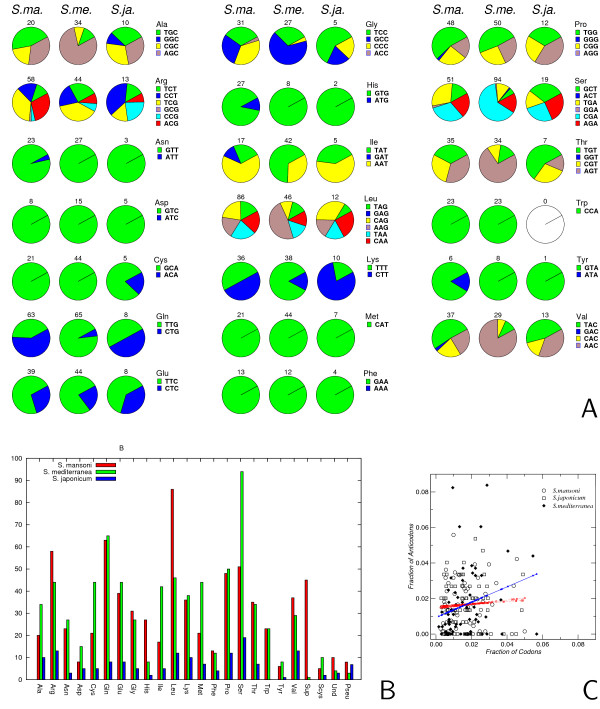
**Comparison of the tRNA complement of *Schistosoma mansoni*, *Schistosoma japonicum*, and *Schmidtea mediterranea***. **A**: Comparison of anti-codon distributions for the 20 amino acids. Numbers below each pie-chart are the total number of tRNA genes coding the corresponding amino acid. Left columns: *S. mansoni*; middle columns: *S. mediterranea*; right columns: *S. japonicum*. **B**: Number of tRNAs encoding a particular amino acid. red: *S. mansoni*, blue: *S. japonicum*, green: *S. mediterranea*. Abbreviations: Sup: putative suppressor tRNAs (CTA, TTA); Scys: Selenocysteine tRNAs (TCA); Pseu: predicted pseudogenes; Und: tRNA predictions with uncertain anticodon; likely these are also tRNA pseudogenes. The Gln-tRNA derived repeat family (see text) is not included in these data. **C**: Comparison of codon usage and anti-codon abundance. No significant correlation is observed for the two schistosomes. For *S. mediterranea *there is a weak, but statistically significant, positive correlation: *t *≈ 2.0

The tRNA complements of the three platyhelminth genomes are compared in detail in Figure 1. The amino acids are represented in approximately equal numbers in *S. mansoni *and *Schmidtea*. Nevertheless, there are several notable deviations. *S. mansoni *contains many more leucine (86 vs. 46) and histidine (27 vs. 8) tRNAs, while serine (51 vs. 94), cysteine (21 vs. 44), methionine (21 vs. 44), and isoleucine (17 vs. 42) are underrepresented. In addition, there are several substantial differences in codon usage. In most cases, *S. mansoni *has a more diverse repertoire of tRNAs: tRNA-Asn-ATT, tRNA-Arg-CGC, tRNA-His-ATG, tRNA-Ile-GAT, tRNA-Pro-GGG, tRNA-Tyr-ATA, tRNA-Val-GAC are missing in *Schmidtea*. Only tRNA-Ser-ACT is present in *Schmidtea *but absent in *Schistosoma*. The tRNA complement of *S. japonicum*, on the other hand, differs strongly from its two relatives. Not only is the number of tRNAs decreased by more than a factor of four, *S. japonicum *also prefers anticodons that are absent or rare in its relatives, such as tRNA-Ala-GGC, tRNA-Cys-ACA, and Lys-CTT. On the other hand, no tRNA-Trp was found. Since the UGG codon is present in many open reading frames we interpret this as a problem with the incompleteness of the genome assembly rather than a genuine gene loss. The reduction in the number of tRNAs is also evident by comparing the number of tRNAs with introns: 27 in *S. mansoni *versus 5 in *S. japonicum*.

It has been shown recently that changes in codon usage, even while coding the same protein sequences, can severely attenuate the virulence of viral pathogens [36] by "de-optimizing" translational efficiency. This observation leads us to speculate that the greater diversity of the tRNA repertoire could be related to the selection pressures of the parasitic life-style of *S. mansoni*. The effect is not straightforward, however, because there is no significant correlation of tRNA copy numbers with the overall codon usage in both *S. mansoni *and *S. japonicum*, Figure 1C. In contrast, a weak but statistically significant correlation can be observed in *Schmidtea mediterranea*. It would be interesting, therefore, to investigate in detail whether there are differences in codon usage of proteins that are highly expressed in different stages of *S. mansoni*'s life cycle, and whether the relative expression levels of tRNAs are under stage-specific regulation.

The most striking result of the tRNAscan-SE analysis was the initial finding of 1,135 glutamine tRNAs (Gln-tRNAs) in *S. mansoni *in contrast to the 8 Gln-tRNAs in *S. japonicum *and 65 in *S. mediterranea*. Nearly all of these (1,098 in *S. mansoni*) were tRNA-Gln-TTG. In addition, an extreme number of 1,824 tRNA-pseudogenes in *S. mansoni *(vs. 951 in *S. japonicum *and 19 in *S. mediterranea*) was predicted. Of these, 1,270 were also homologous to tRNA-Gln-TTG. These two groups of tRNA-Gln-TTG-derived genes (those predicted to be pseudogenes and those predicted to be functional tRNAs) totaled 2,368. These high numbers suggest a tRNA-derived mobile genetic element. We therefore ran the 2,368 *S. mansoni *tRNA-Gln-TTG genes through the RepeatMasker program [37]. Almost all of them (2,342) were classified as SINE elements. Further BLAST analysis revealed that these elements are similar to members of the Sm-*α *family of *S. mansoni *SINE elements [38]. Removal of these SINE-like elements yielded a total of 63 predicted glutamine-encoding tRNAs in *S. mansoni*. About 650 of 951 pseudogenes in *S. japonicum *derived from tRNA-Pro-CGG.

Homology-based analysis yielded similar, though somewhat less sensitive, results to those of tRNAscan-SE. For instance, a BLAST search in *S. mansoni *with Rfam's tRNA consensus yielded 617 predicted tRNAs compared to the 663 predictions made by tRNAscan-SE.

### Ribosomal RNAs

As usual in eukaryotes, the 18S, 5.8S, and 28S genes are produced by RNA polymerase I from a tandemly repeated polycistronic transcript, the ribosomal RNA operon. The *S. mansoni *genome contains about 90-100 copies [39,40] which are nearly identical at sequence level, because they are subject to concerted evolution [41]. The repetitive structure of the rRNA operons causes substantial problems for genome assembly software [42]. In order to obtain a conservative estimate of the copy number, we retained only partial operon sequences that contained at least two of the three adjacent rRNA genes. We found 48 loci containing parts of 18S, 5.8S, and 28S genes, 32 loci covering 18S and 5.8S rRNA, and 57 loci covering 5.8S and 28S rRNAs [see Additional file 1 - Figures S1 and S2]. Adding the copy numbers, we have not fewer than 80 copies (based on linked 18S rRNAs) and no more than 137 copies (based on linked 5.8S rRNA). The latter is probably an overestimate due to the possibility that the 5.8S rRNA may be contained in two scaffolds. The copy number of rRNA operons is thus consistent with the estimate of 90-100 from hybridization analysis [39]. An analogous analysis of the current *S. japonicum *assembly yields less accurate results. Due to the many short fragments, we obtained 90 copies; the true number may lie between 50 and 280, however.

The 5S rRNA is a polymerase III transcript that has not been studied in schistosomes so far. We found 21 copies of the 118 nt long 5S rRNA in *S. mansoni*, compared with 13 copies in *S. japonicum*. Four of the 21 copies are located within a 3,000 nt cluster on *Scaffold010519*.

### Spliceosomal RNAs and Spliced Leader RNA

**Figure 2:**
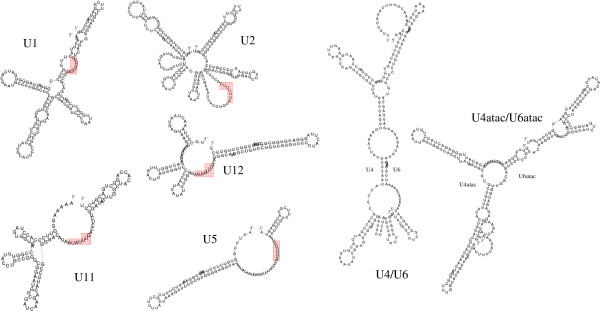
**Secondary structures of the nine snRNAs and the interaction complexes of U4/U6 and U4atac/U6atac, respectively, in *S. mansoni***. Structure prediction was performed by RNAfold, RNAalifold and for U4/U6 and U4atac/U6atac by RNAcofold from the RNA Vienna Package [96,108]. Boxes indicate Sm binding sites. Additional details on sequences, structures, and alignments are available at the supplementary material.

Spliceosomes, the molecular machines responsible for most splicing reactions in eukaryotic cells, are ribonu-cleoprotein complexes similar to ribosomes [43]. The major spliceosome, which cleaves GT-AG introns, includes the five snRNAs U1, U2, U4, U5, and U6. In the *S. mansoni *genome, all of them are multicopy genes. By homology search we found 34 U1, 15 U2, 19 U4, 9 U5, and 55 U6 sequences in the genome assembly. Interpreting all sequences that are identical in short flanking regions as the same, we would retain only 3 U1, 3 U2, 1 U4, 2 U5, and nine U6 genes [44]. The true copy number in the *S. mansoni *genome is most likely somewhere between these upper and lower bounds. For *S. japonicum*, the corresponding numbers are U1: 2-6, U2: 1-63 U2, U4: 1-6 U4, U5: 1-24, and U6: 2-12. Due to the more fragmented genome assembly we expect the true numbers to be closer to the lower bounds. Secondary structures for these candidates are similar to those of typical snRNAs, Figure 2.

A second, much less frequent, minor spliceosome is responsible for the processing of atypical AT-AC introns. It shares only the U5 snRNA with the major spliceosome. The other four RNA components are replaced by variants called U11, U12, U4atac, and U6atac [45]. The minor-spliceosomal snRNAs are typically much less conserved than the RNA components of the major spliceosome [44]. It was not surprising, therefore, that these RNAs were detectable only by means of GotohScan[8] but not with the much less sensitive BLAST searches. Although U4atac and U6atac are quite diverged compared to known homologs, they can be recognized unambiguously based on both secondary structure and conserved sequence motifs. Furthermore, the U4atac and U6atac sequences can interact to form the functionally necessary duplex structure shown in Figure 2. As in many other species, there is only a single copy of each of the minor spliceosomal snRNAs in both of the schistosome genomes, Tab. 1. An analysis of promoter sequences showed that the putative snRNA promoter motifs in *S. mansoni *are highly derived. Only one of the two U12 genes exhibited a clearly visible snRNA-like promoter organization.

The Spliced Leader (SL) RNA is one of the very few previously characterized ncRNAs from *S. mansoni *[26]. The 90 nt SL RNA, which was found in a 595 nt tandemly repeated fragment (accession number M34074), contains the 36 nt leader sequence at its 5' end which is transferred in the *trans*-splicing reaction to the 5' termini of mature mRNAs. Using blastn, we identified 54 SL RNA genes. These candidates, along with 100 nt flanking sequence, were aligned using ClustalX, revealing 6 sequences with aberrant flanking regions, which we suspect to be pseudogenic. The remaining sequences are 43 identical copies and 5 distinct sequence variants. A secondary structure analysis corroborates the model of [26], according to which the *S. mansoni *SL RNA has only two loops, with an unpaired Sm binding site [see Additional File 1 - Figure S3]. This coincides with the SL RNA structure of Rotifera [46], but is in contrast to the SL RNAs in most other groups of eukaryotes, which exhibit single or triple stem-loop structures [47]. A blastn-search against *S. mansoni *EST data confirms that the 5' part of the SL is indeed *trans*-spliced to mRNAs. Several nearly identical SL RNA homologs are found in *S. japonicum*.

### SRP RNA and Ribonuclease P RNA

Signal recognition particle (SRP) RNA, also known as 7SL RNA, is part of the signal recognition particle, a ribonucleoprotein that directs packaged proteins to their appropriate locations in the endoplasmic reticulum. Although one of the protein subunits of this ribonucleoprotein was cloned in 1995 [48], little is known about the other subunits or the RNA component in *S. mansoni*. We found eight probable candidates for the SRP RNA, with one almost canonical sequence [see Additional file 1 - Figure S4], and four possible candidates with point mutations which may influence their function.

The RNA component of Ribonuclease P (RNase P) is the catalytically active part of this enzyme that is required for the processing of tRNA precursors [49,50]. We found one classic RNase P RNA in the *S. mansoni *genome using both GotohScan and rnabob with the eukaryotic ("nuclear") Rfam consensus sequence for RNase P as search sequence.

### MicroRNAs

MicroRNAs are small RNAs that are processed from hairpin-like precursors, see e.g. [51]. They are involved in post-transcriptional regulation of mRNA molecules. So far, no microRNAs have been verified experimentally in *S. mansoni*. The presence of four protein-coding genes encoding crucial components of the microRNA processing machinery (Dicer, Argonaut, Drosha, and Pasha/DGCR8) [52,53], and the presence of Argonaut-like genes in both *S. japonicum *[54] and *S. mansoni *(detected by tblastn in EST data, see Supplemental Data online), strongly suggests that schistosomes have a functional microRNA system. Indeed, most recently five miRNAs were found by direct cloning in *S. japonicum *that are also conserved in *S. mansoni *[55]: *let-7*, *mir-71*, *bantam*, *mir-125*, and a single schistosome-specific microRNA. These sequences, including the precursor hairpins, are well conserved in *S. japonicum*. On the other hand, the microRNA precursor sequences of both schistosomes are quite diverged from the consensus of the homologous genes in Bilateria.

**Figure 3:**
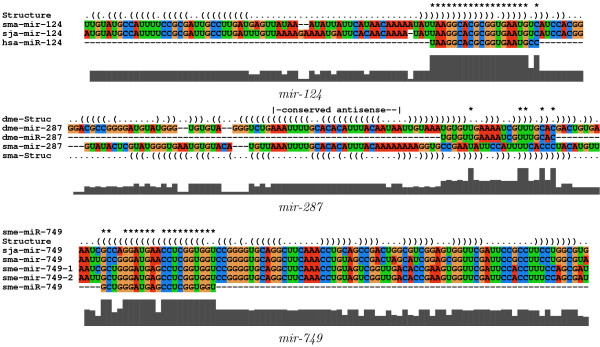
**Multiple sequence alignments of the *pre*-miRNAs that were computationally found in *S. mansoni***. For *mir-124 *and *mir-749 *the sequences share a common consensus structure. The uncertain *mir-287 *candidate clusters together with *mir-124 *in insect genomes. However, though it also exhibits a single stem-loop structure, it is different from that of insects. Here the sequence is only conserved at the antisense region of the annotated mature miRNA.

Using bioinformatics (see methods) we were able to find only one further miRNA candidate in *S. mansoni*, *mir-124*, that is also conserved in *S. japonicum*. In insects, this miRNA is clustered with *mir-287*. The distance of both miRNAs is approximately 8 kb in Drosophilids. We found an uncertain *mir-287 *candidate in *S. mansoni*, however, on a different scaffold than *mir-124*. Although this sequence nicely folds into a single stem-loop structure, it is conserved only antisense to the annotated mature sequence in insects (see, Figure 3). This *S. mansoni mir-287 *candidate does not seem to be conserved in *S. japonicum*.

In [56], 71 microRNAs are described for the distantly related trematode *Schmidtea mediterranea*, and additional ones are announced in a recent study focussing on piRNAs [57]. The overwhelming majority, 54, were reported to be members of 29 widely conserved metazoan microRNA families, although in some cases even the mature miRNA sequence is quite diverged. Therefore, we regard several family assignments as tentative at best. Of those 29 miRNAs, we found *mir-124 *only. However, the schistosome sequences are more related to the other bilaterian *mir-124 *homologs than to those of *S. mediterranea*. Out of the remaining 54 miRNAs that were annotated in *S. mediterranea *we found that *mir-749 *is also conserved in the two schistosome species. Here, the sequences show a common consensus sequence *and *secondary structure in their precursors (see Figure 3).

The small number of recognizable microRNAs in schistosomes is in strong contrast to the extensive microRNA complement in *S. mediterranea*, indicating massive loss of microRNAs relative to the planarian ancestor. This may be a consequence of the parasitic lifestyle of the schistosomes.

### Small Nucleolar RNAs

Small nucleolar RNAs play essential roles in the processing and modification of rRNAs in the nucleolus [58,59]. Both major classes, the box H/ACA and the box C/D snoRNAs are relatively poorly conserved at the sequence level and hence are difficult to detect in genomic sequences. This has also been observed in a recent ncRNA annotation project of the *Trichoplax adhaerens *genome [8]. The best-conserved snoRNA is the atypical U3 snoRNA, which is essential for processing of the 18S rRNA transcript into mature 18S rRNA [60]. In the current assembly of the *S. mansoni *genome we found six U3 loci, but they are also identical in the flanking sequences, suggesting that in fact there is only a single U3 gene. No unambiguous homologue was detected for any of the other known snoRNAs.

A *de novo *search for snoRNAs (see methods for details) resulted in 2,610 promising candidates (1,654 box C/D and 956 box H/ACA), see Supplemental Data online. All these predictions exhibit highly conserved sequence boxes as well as the typical secondary features of box C/D and box H/ACA snoRNAs, respectively.

A comparison of the predicted snoRNAs with the entries in the Rfam[61] and NONCODE[62] databases returned only 47 hits that match to several other RNAs like tRNAs, parts of the rRNA operon, snRNAs, mRNAlike genes and a few of our candidates map to the hammerhead ribozyme. These sequences are likely false positives and have been removed from the candidate list. The number of predicted candidates is much larger than the number of snoRNAs reported in other organisms; for instance [59] lists 456 for the human genome. Although we most likely do not yet know the full snoRNA complement of eukaryotic genomes, we have to expect that a large fraction of prediction will turn out to be false positives.

We therefore analysed the conservation of the candidates in *S. japonicum *and focussed on the snoRNA candidates with targets in the 18S, 28S and/or 5.8S ribosomal RNA. While targets are predicted for more than half of the candidates, see Table 2, the numbers are drastically reduced when conservation of the candidates in *S. japonicum *is required. Note, furthermore, that the fraction of conserved candidates is strongly enriched among those with ribosomal RNA targets, indicating that these sets are likely to contain a sizeable fraction of true positives. This filtering step leaves us with 227 box C/D and 352 box H/ACA snoRNA candidates. While still high, these numbers fall into the expected range for a metazaon snoRNA complement.

**Table 2:** Conservation and target prediction of snoRNA candidates.

**snoReport****targets**	**Box C/D **(**snoscan**)			**Box H/ACA **(**RNAsnoop**)		
	≥ 2	1	0	≥ 2	1	0
predicted in *S. mansoni*	926	110	613	284	495	177
conserved in *S. japonicum*	200	27	83	149	203	62

Only ribosomal RNAs were searched for putative target sites.

We remark, finally, that five of the snoRNA candidates (three box C/D and two box H/ACA) are also conserved in *Schmidtea mediterranea*.

### Other RNA motifs

Two examples of relatively well-known schistosome non-coding RNAs are the hammerhead ribozyme motifs within the Sm-*α *and Sj-*α *SINE-like elements [27,28]. A blastn search of the hammerhead ribozyme motif from the Rfam database resulted in ~38,500 candidates for *S. mansoni *in contrast to ~5,000 candidates for *S. japonicum*. While high, this number is not surprising considering the generally high copy number of SINE elements; previously, the copy number for Sm-*α *elements in the *S. mansoni *genome was estimated to exceed 10,000 [27]. The highly conserved potassium channel RNA editing signal [63,64] is another structured RNA element that was described previously [65]. We found nine copies of this hairpin structure in the *S. mansoni *genome assembly and three in *S. japonicum*.

### Uncertain and missing candidates

Both the MRP RNA [2,3,66] and the 7SK RNA [4,5,67] have highly variable, rapidly evolving sequences that make them difficult or impossible to detect in invertebrate genomes. Their ancient evolutionary origin and their extremely conserved molecular house-keeping functions make it more than likely that they are present in the schistosome genomes as well. In both cases, we have not been able to identify unambiguous homologs. There are, however, plausible candidates. We briefly describe them in the following paragraphs since they may warrant further attention and may be a useful starting point for subsequent experimental studies, as exemplified by the history of discovery of the snRNA in *Giardia intestinalis *[68-70].

MRP RNA has multiple functions, among them mitochondrial RNA processing and nucleolar pre-rRNA processing. The *S. mansoni *MRP candidate fits the general secondary structure model of metazoan MRP RNAs [2,3,66] and analysis with RNAduplex shows that the candidate contains a pseudoknot which exhibited striking sequence identity with known MRPs. The locus is well-conserved in *S. japonicum*. On the other hand, stems 1 and 12 were divergent compared to known MRPs, and stem 19 also fails to display clear similarities with known MRPs. Although quite likely a true MRP homolog, we therefore consider this sequence only tentative.

7SK RNA is a general transcriptional regulator, repressing transcript elongation through inhibition of transcription elongation factor PTEFb and also suppresses the deaminase activity of APOBEC3C [71]. The *S. mansoni *7SK candidate has a 5' stem similar to that described in other invertebrates [5], and parts of the middle of the sequence are also recognizable. There is, furthermore, a homologous locus in the genome of *S. japonicum*. However, the 3' stem (which was followed by a poly-T terminator) was not conserved. In addition, a large sequence deletion was evident.

Three major classes of ncRNAs were expected, but not found, in the *S. mansoni *genome. As in all other invertebrates genomes, no candidate sequence was found for a telomerase RNA. *S. mansoni *almost certainly has a canonical telomerase holoenzyme, since it encodes telomerase proteins (Smp_066300 and Smp_066290) and has the same telomeric repeat sequences as many other metazoan animals [72]. Telomerase RNAs are notoriously difficult to find, as they are highly divergent among different species, varying in both size and sequence composition [7,73]. Vault RNAs are known in all major deuterostome lineages [74], and homologs were recently also described in two lophotrochozoan lineages [75]. Since *S. mansoni *has a homolog of the major vault protein (Smp_006740) we would also expect a corresponding RNA component to be present. So far, Y RNAs have been found only in vertebrates [76,77] and in nematodes [78,79], although the Ro RNP, that they are associated with, seems to be present in most or even all eukaryotes.

## Conclusion

We have described here a detailed annotation of "housekeeping" ncRNAs in the genomes of the parasitic platyhelminth *Schistosoma mansoni *and *Schistosoma japonicum*. Limited to the best conserved structured RNAs, our work nevertheless uncovered important genomic features such as the existence of a SINE family specific to *Schistosoma mansoni*, which is derived from tRNA-Gln-TTG. Our data furthermore establish the presence of a minor spliceosome in schistosomes and confirms spliced leader *trans*-splicing. With a coverage of at least 90-95% of the genomic DNA, missing data are not a significant problem. The fragmented genome assemblies, however, preclude accurate counts of the multi-copy genes.

Platyhelminths are known to be a fast-evolving phylum [80]. It is not surprising therefore that in particular the small ncRNAs are hard or impossible to detect by simple homology search tools such as blastn. Even specialized tools have been successful in identifying only the better conserved genes such as tRNA, microRNAs, RNase P RNA, SRP RNA. The notoriously poorly conserved families, such as snoRNAs, telomerase RNA, or vault RNAs, mostly escaped detection.

The description of several novel, and in many cases quite derived, schistosome ncRNAs contributes significantly to the understanding of the evolution of the corresponding RNA families. The schistosome ncRNA sequences, furthermore, are an important input to subsequent homology search projects, since they allow the construction of improved descriptors for sequence/structure-based search algorithms. Last but not least, the ncRNA annotation tracks are an important contribution to the genome-wide annotation datasets of both *S. mansoni *and *S. japonicum*. It not only contributes the protein-based annotation but also helps to identify annotation errors, e.g. cases where putative proteins are annotated that overlap rRNA operons or other ncRNAs.

The house-keeping ncRNAs considered in this study are almost certainly only the proverbial tip of the platyhelminth ncRNAs iceberg. The discovery of a large number of mRNA-like ncRNAs (mlncRNAs) in many eukaryotes (compiled e.g. in the RNAdb[81] and reviewed e.g. in [1]), and in particular in many other invertebrate species (nematodes [82], insects [83,84]) suggests that similar transcripts will also be abundant in schistosomes. The abundant EST data for both schistosome species [85,86] can provide a starting point e.g. for an analysis along the lines of [87]. Computational surveys, furthermore, have provided evidence for large numbers of RNAs with conserved secondary structures in other invertebrates [88-90]. The underlying methods, such as RNAz[91], are inherently comparative, presenting difficulties for application to schistosome genomes due to the large evolutionary distance between schistosome and non-schistosome genomes. This is also the case for a recent approach to identify mRNA-like non-coding RNAs with very low levels of sequence conservation based on their intron structure [92]. A deeper understanding of the non-coding transcriptome of schistosomes will therefore have to rely primarily on experimental approaches, either by means of tiling arrays or by means of high throughput transcriptome sequencing.

## Methods

### tRNA annotation

We used tRNAscan-SE[93] with default parameters to annotate putative tRNA genes. As additional confirmation, the genome sequence was searched using tRNA consensus sequences from the Rfam database [61]. In order to obtain suitable data for comparison, the genome of the free-living platyhelminth *Schmidtea mediterranea *[17] was searched alongside that of *S. mansoni *and *S. japonicum*.

### microRNA annotation

We followed the general protocol outlined in [8] to identify miRNA precursors, using all metazoan miRNAs listed in miRBase [94] [Release 11.0, http://microrna.sanger.ac.uk/sequences/]. The initial search was conducted by blastn with *E *< 0.01 with the mature and mature* miRNAs as query sequences. The resulting candidates were then extended to the length of the precursor sequence of the search query and aligned to the precursors using ClustalW[95]. Secondary structures were predicted using RNAfold[96] for single sequences and RNAalifold[97] for alignments. Candidates that did not fold into miRNA-like hairpin structures were discarded. The remaining sequences were then examined by eye to see if the mature miRNA was well-positioned in the stem portion of each putative precursor sequence. In addition, we used the final candidates to search the *S. japonicum *and *S. mediterranea *genomes to examine whether these sequences are conserved in Schistosoma and/or Platyhelminthes.

### snoRNA annotation

We compared all the known human and yeast snoRNAs that are annotated in the snoRNAbase[98] to the *S. mansoni *genome using BLAST[99] and GotohScan[8]. The search for novel snoRNA candidates was performed only on sequences that were not annotated as protein-coding or another ncRNA in the current *S. mansoni *assembly. The SnoReport program [100] was used to identify putative box C/D and box H/ACA snoRNAs on both strands. Only the best predictions, i.e., those that show highly conserved boxes and canonical structural motifs, were kept for further analysis. The remaining candidates are further analysed for possible target interactions with ribosomal RNAs using snoscan[101] for box C/D and RNAsnoop[102] for box H/ACA snoRNA candidates. In addition, the sequences were checked for conservation in *S. japonicum *and *S. mediterranea *using BLAST. To estimate the number of false predictions we compared the candidate snoRNAs with common ncRNA databases, in particular Rfam[61] and NONCODE[62]. All sequences that match a non-snoRNA ncRNA were discarded.

### Other RNA families

For other families, we employed the following five steps:

(a) For candidate sequences of ribosomal RNAs, spliceosomal RNAs, the spliced leader (SL) and the SRP RNA, we performed BLAST searches with *E *< 0.001 using the known ncRNA genes from the NCBI and Rfam databases. For the snRNA set, see [44]. For 7SL RNA we used ***X04249***, for 5S and 5.8S rRNAs we used the complete set of Rfam entries, for the SSU and LSU rRNAs, we used ***Z11976 ***and ***NR_003287***, respectively. The SL RNAs were searched using SL RNA entries from Rfam and the sequences reported in [26]. For more diverged genes such as minor snRNAs, RNase MRP, 7SK, and RNase P, we used GotohScan[8], an implementation of a full dynamic programming alignment with affine gap costs. In cases where no good candidates were found we also employed descriptor-based search tools such as rnabob http://selab.janelia.org/software.html.

(b) In a second step, known and predicted sequences were aligned using ClustalW[95] and visualized with ClustalX[103]. To identify functional secondary structure, RNAfold, RNAalifold, and RNAcofold[104] were used. Combined primary and secondary structures were visualized using stockholm-format alignment files in the emacs editor utilizing ralee mode [105]. Alignments are provided at the Supplemental Data online.

(c) Putatively functional sequences were distinguished from likely pseudogenes by analysis of flanking genomic sequence. To this end, the flanking sequences of snRNA and SL RNA copies were extracted and analyzed for conserved sequence elements using MEME[106]. Only snRNAs with plausible promoter regions were reported.

(d) Additional consistency checks were employed for individual RNA families, including phylogenetic analysis by neighbor-joining [107] to check that candidate sequences fall at phylogenetically reasonable positions relative to previously known homologs. For RNase MRP RNA candidates, RNAduplex http://www.tbi. univie.ac.at/RNA/RNAduplex.html was used to find the pseudoknot structure. In order to confirm that the SL RNA candidate is indeed *trans*-spliced to mRNA transcripts, we searched the *FAPESP Genoma Schistosoma mansoni *website for ESTs including fragments of the predicted SL RNA. We found 52 ESTs with blastn*E *< 0.001 that span the predicted region of the SL RNA (nt 8-38), indicating that this RNA does indeed function as a spliced leader.

(e) Accepted candidate sequences were used as BLAST queries against the *S. mansoni *genome to determine their copy number in the genome assembly.

### Additional Data Online

The website http://www.bioinf.uni-leipzig.de/Publications/SUPPLEMENTS/08-014 provides extensive machine readable information, including sequence files, alignments, and genomic coordinates.

## Authors' contributions

CSC, PB, and PFS designed the study. CSC, MM, DR, JH, CBS, SK, CSA, and PFS performed the computational analyses. CSC wrote the first draft of the manuscript. All authors contributed to the final assessment of the data as well as the writing of the final version of the manuscript. CSC, MM, DR, JH should be considered as joint first authors.

## Supplementary Material

Additional file 1 **Supplemental figures and captions**. contains supplemental Figures S1 - S4 mentioned in the main text. Click here for file
