# Supplementary material for: Homology-based annotation of non-coding RNAs in the genomes of Schistosoma mansoni and Schistosoma japonicum
Source: BMC Genomics. 2009 Oct 8;10:464. doi: 10.1186/1471-2164-10-464 (PMC2770079; doi:10.1186/1471-2164-10-464)
Supplement: Additional file 1 — Supplemental figures and captions. contains supplemental Figures S1 - S4 mentioned in the main text. [file 1471-2164-10-464-S1.PDF]

Non-coding RNA Annotation of the Schistosoma mansoni Genome (SUPPLEMENT)

Claudia S. Copeland[1,2] , Manja Marz[1] , Dominic Rose[1] , Jana Hertel[1] , Paul J. Brindley[2] , Clara Bermudez Santana[1,8] , Stephanie Kehr[1] , Camille Stephan-Otto Attolini[3] , Peter F. Stadler[1,4,5,6,7,*]

[1]Bioinformatics Group, Department of Computer Science, and Interdisciplinary Center for Bioinformatics, University of Leipzig, Härtelstraße 16-18, D-04107 Leipzig, Germany
[2]Department of Microbiology, Immunology & Tropical Medicine, George Washington University Medical Center, 2300 I Street, NW, Washington, DC 20037, USA
[3]Memorial Sloan-Kettering Cancer Center, Computational Biology Department, 1275 York Avenue, Box # 460, New York, NY 10065, USA
[4]Max Planck Institute for Mathematics in the Sciences, Inselstrasse 22, D-04103 Leipzig, Germany
[5]Fraunhofer Institute for Cell Therapy and Immunology, Perlickstraße 1, D-04103 Leipzig, Germany
[6]Santa Fe Institute, 1399 Hyde Park Rd., Santa Fe, NM 87501, USA
[7]Institute for Theoretical Chemistry, University of Vienna, Währingerstraße 17, A-1090 Wien, Austria
[8]Department of Biology, National University of Colombia, Carrera 45 No. 26-85, Bogota, D.C., Colombia

Email: Peter F. Stadler - studla@bioinf.uni-leipzig.de;

*Corresponding author

References

1. Rajkovic A, Davis RE, Simonsen JN, Rottman FM: **A spliced leader is present on a subset of mRNAs from the human parasite *Schistosoma mansoni***. *Proc Natl Acad Sci USA* 1990, **87**:8879–8883.

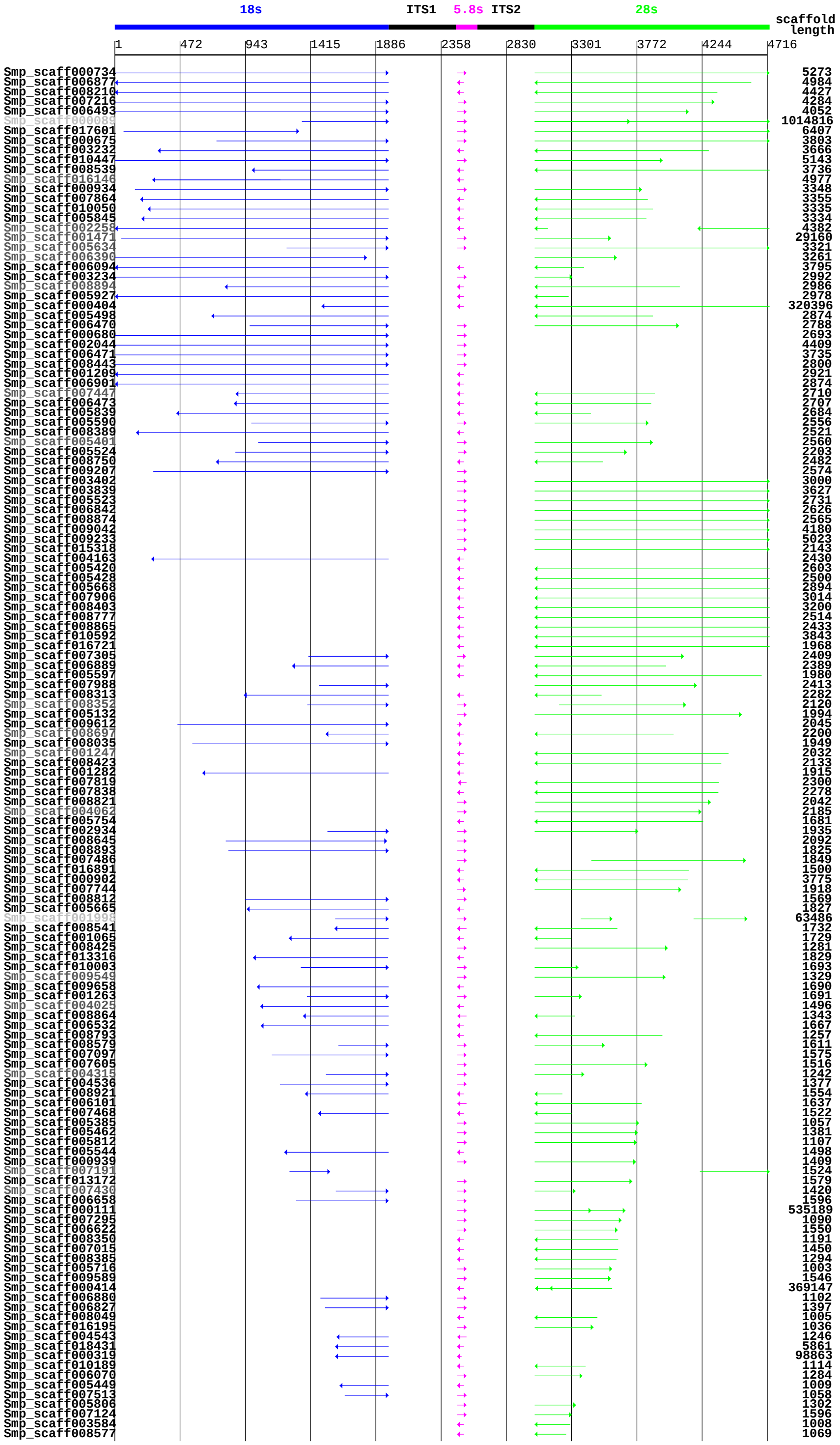

Figure 1: **Fragments of RNA Operons.**
Whole and partial pol-I transcribed rRNA operons. Scale representation of portions of scaffolds that include either whole rRNA operons or fragments including 18S and 5.8S or 28S and 5.8S. Right-facing arrows represent plus-strand transcripts; left-facing arrows represent minus-strand transcripts. Scaffold names are shown in the far left column; names in light gray have large runs of unknown nucleotides ("N's"), names in dark gray have smaller runs of unknown nucleotides. Scaffold lengths are shown in the far right column. Top line: scale drawing of the whole pol-I transcribed rRNA operon. Second line: scale, in nt. Scaffold lines: blue arrows: 18S regions, pink arrows: 5.8S regions, green arrows: 28S regions.

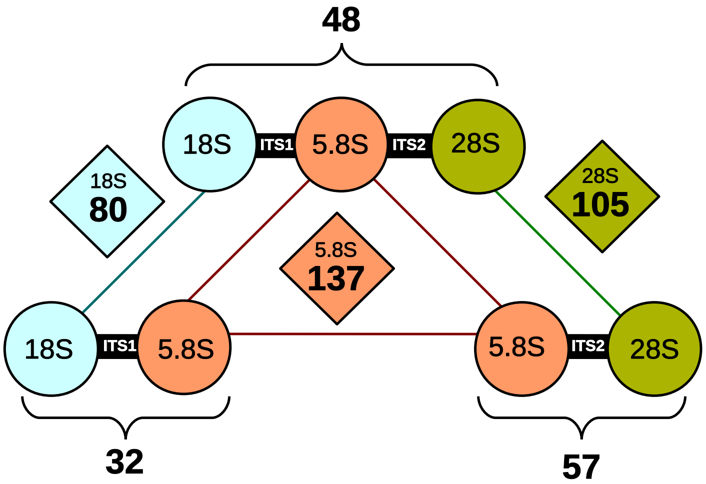

Figure 2: **rRNA subunits.**
Copy numbers of pol-I transcribed rRNA subunits found associated with larger parts of the rRNA operon. Subunits of the ribosomal operon are represented as colored circles (18S: blue, 5.8S: orange, 28S: green). Copy numbers of the whole operon, 5' end, and 3' end are shown in brackets above or below the representations of the rRNA genes for these regions. Total copy number of each subunit type are shown in diamonds colored to match their respective subunits (18S: blue, 5.8S: orange, 28S: green).

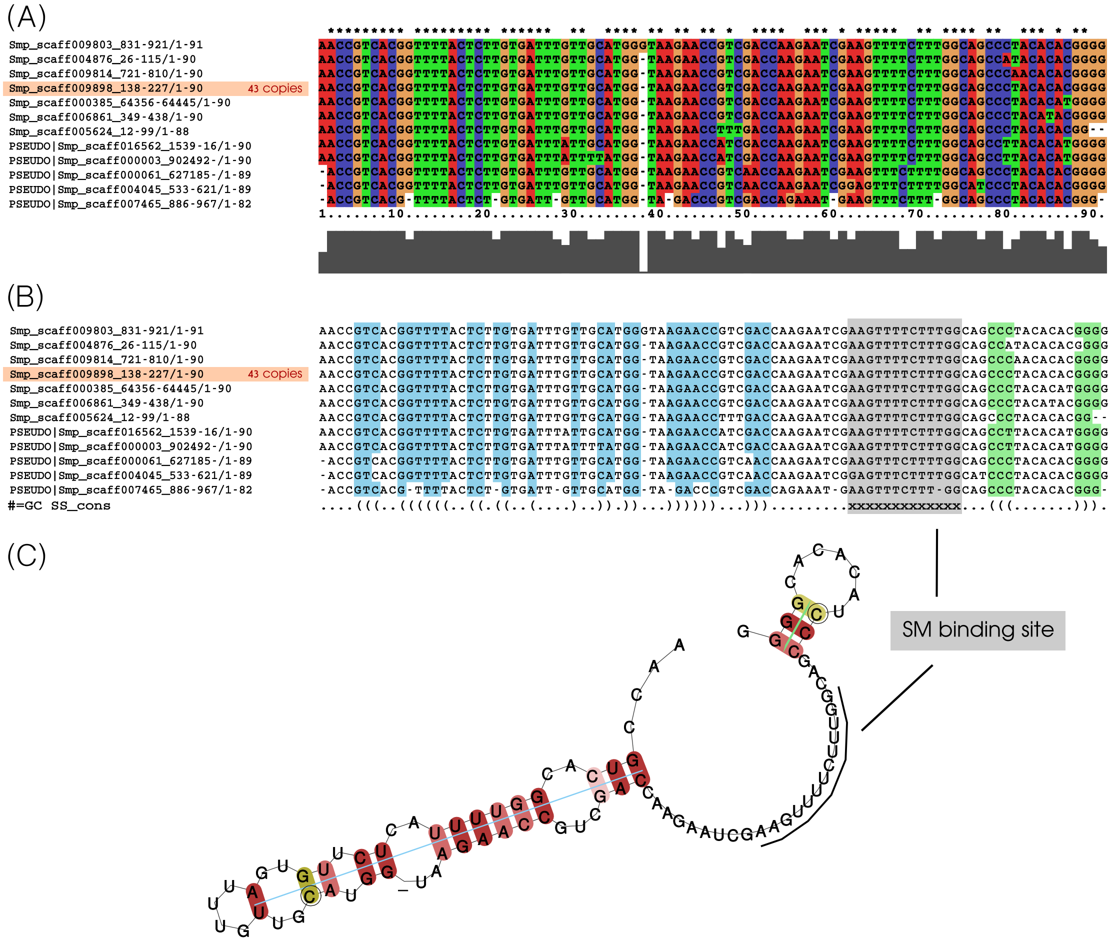

Figure 3: **SL RNA sequences and structure.**
A) Clustal alignments of SL RNA candidates and putative pseudogenes. All sequences are single-copy except for the salmon- highlighted sequence, which represents a cluster of 43 copies. B) Alignments in emacs ralee mode, with structural elements highlighted. Consensus secondary structure is represented at the last line of the alignment. Blue and green highlight: base-paired regions. Grey highlight: the Sm binding site. C) Secondary structure predicted by RNAalifold with the constraint that the Sm binding site must be unpaired. For a full alignment, including flanking regions, see http://www.bioinf.uni-leipzig.de/Publications/SUPPLEMENTS/08-014. Secondary structure analysis of these candidates revealed structural conservation and thermodynamic stability indicating a likely ncRNA. Like [1] we found that the *S. mansoni* SL RNA has only two loops, with an unpaired Sm binding site, whereas most other SL RNAs have a triple stem-loop structure.

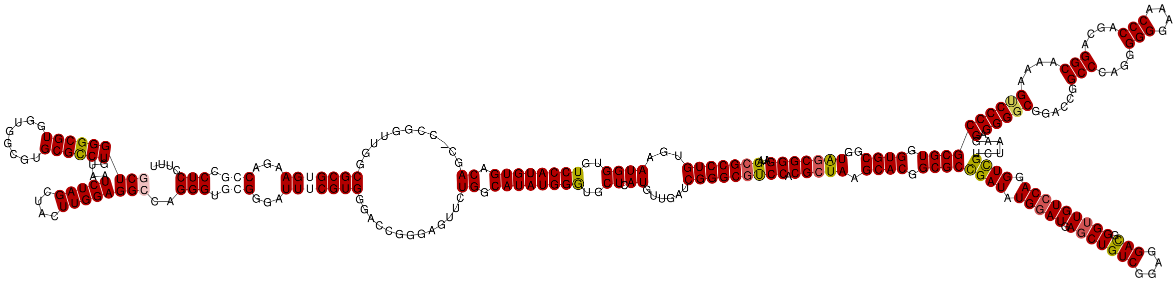

Figure 4: **SRP RNA.**
Secondary structure for the predicted *S. mansoni* signal recognition particle. Red nucleotides represent base pairs with conserved nucleotides across different species in the alignment. Yellow nucleotides represent positions with a high level of point mutations in different species, but with conserved secondary structure (compensatory mutations). Alignments are also available as supplementary files.
